# 3017- Basic and clinical immunology – 3017: A functional polymorphism in IL-5 receptor alpha may influence asthma severity in patients with aspirin-exacerbated respiratory disease

**DOI:** 10.1186/1939-4551-6-S1-P193

**Published:** 2013-04-23

**Authors:** Purevsuren Losol, Seung-Hyun Kim, Yoo Seob Shin, Young Min Ye, Hae-Sim Park

**Affiliations:** 1 Allergy and Clinical Immunology, Ajou University School of Medicine, Suwon, South Korea

## Background

Eosinophilic infiltration into the tissues of the airway is a central feature of aspirin-exacerbated respiratory disease (AERD). Eosinophil activation and survival are profoundly influenced by IL-5 and its receptor, IL-5R. In patients susceptible to allergic disorders, *IL5RA* polymorphisms have been reported, however, an association with AERD remains unclear. We hypothesise that the presence of *IL5RA* polymorphisms will increase the genetic susceptibility to AERD.

## Methods

We recruited 139 AERD patients, 171 aspirin-tolerant asthma (ATA) patients and 160 normal controls. *IL5RA* polymorphisms (-5993G>A, -5567C>G, -5091G>A) were genotyped and functional studies were assessed by luciferase reporter assay and electrophoretic mobility shift assay (EMSA). Asthma severity was classed into three groups according the FEV1 predicted value at the enrolment period following the GINA guidelines.

## Results

The genotype frequency of -5993G>A was significantly associated with asthma severity in AERD patients (*P*<0.05). The frequency of the minor allele at the *IL5RA* -5993G>A polymorphism was significantly higher in moderate and severe patients when compared with mild patients (severe *vs*. mild, *P*=0.032 for the dominant model; severe *vs*. moderate, *P*=0.041 for the co-dominant model, and *P*=0.012 for the dominant model). Moderate and severe patients in the AERD group, carrying the AA genotype at -5993G>A, had a significantly higher prevalence of specific IgE to staphylococcal superantigens than those with the GG/GA genotype (*P*=0.005). *In vitro*, the -5993A allele had a higher promoter activity compared to the -5993G allele in human mast cells (HMC-1) (*P*=0.030) and eosinophilic cells (HL-60) (*P*=0.013). In EMSA, a -5993A probe produced a specific shifted band than the -5993G had. The shifted band produced by the -5993A probe was not visible in the presence of the nonlabeled -5993G probe but remained visible in the presence of the nonlabeled -5993A probe.

## Conclusions

A functional polymorphism in *IL5RA* could contribute to eosinophil and mast cell activation in AERD, and aggravate asthma severity along with specific IgE responses to staphylococcal superantigens.

